# In-Advance Prediction of Pressure Ulcers via Deep-Learning-Based Robust Missing Value Imputation on Real-Time Intensive Care Variables

**DOI:** 10.3390/jcm13010036

**Published:** 2023-12-20

**Authors:** Minkyu Kim, Tae-Hoon Kim, Dowon Kim, Donghoon Lee, Dohyun Kim, Jeongwon Heo, Seonguk Kang, Taejun Ha, Jinju Kim, Da Hye Moon, Yeonjeong Heo, Woo Jin Kim, Seung-Joon Lee, Yoon Kim, Sang Won Park, Seon-Sook Han, Hyun-Soo Choi

**Affiliations:** 1 Department of Research & Development, Ziovision Co., Ltd., Chuncheon 24341, Republic of Korea; minkyu.kim@ziovision.co.kr (M.K.); dakim1204@ziovision.co.kr (D.K.); dannylee@ziovision.co.kr (D.L.); dohyeon.kim@ziovision.co.kr (D.K.); 2 Department of Internal Medicine, Kangwon National University, Chuncheon 24341, Republic of Korea; blessing0104@naver.com (T.-H.K.); doctorhjw@naver.com (J.H.); kjj451007@naver.com (J.K.); ansekgo@naver.com (D.H.M.); yonjong1954@naver.com (Y.H.); pulmo2@kangwon.ac.kr (W.J.K.); medfman@kangwon.ac.kr (S.-J.L.); 3 Department of Convergence Security, Kangwon National University, Chuncheon 24341, Republic of Korea; uk99@knuh.or.kr; 4 Biomedical Research Institute, Kangwon National University Hospital, Chuncheon 24289, Republic of Korea; xvop11@naver.com; 5 Department of Pulmonology, Kangwon National University Hospital, Chuncheon 24289, Republic of Korea; 6 Department of Computer Science and Engineering, Kangwon National University, Chuncheon 24341, Republic of Korea; yooni@kangwon.ac.kr; 7 Department of Medical Informatics, School of Medicine, Kangwon National University, Chuncheon 24341, Republic of Korea; chicwon229@kangwon.ac.kr; 8 Institute of Medical Science, School of Medicine, Kangwon National University, Chuncheon 24341, Republic of Korea; 9 Department of Computer Science and Engineering, Seoul National University of Science and Technology, Seoul 01811, Republic of Korea

**Keywords:** intensive care unit, clinical decision support systems, pressure ulcers, time series, missing values, machine learning, deep learning

## Abstract

Pressure ulcers (PUs) are a prevalent skin disease affecting patients with impaired mobility and in high-risk groups. These ulcers increase patients’ suffering, medical expenses, and burden on medical staff. This study introduces a clinical decision support system and verifies it for predicting real-time PU occurrences within the intensive care unit (ICU) by using MIMIC-IV and in-house ICU data. We develop various machine learning (ML) and deep learning (DL) models for predicting PU occurrences in real time using the MIMIC-IV and validate using the MIMIC-IV and Kangwon National University Hospital (KNUH) dataset. To address the challenge of missing values in time series, we propose a novel recurrent neural network model, GRU-D++. This model outperformed other experimental models by achieving the area under the receiver operating characteristic curve (AUROC) of 0.945 for the on-time prediction and AUROC of 0.912 for 48h in-advance prediction. Furthermore, in the external validation with the KNUH dataset, the fine-tuned GRU-D++ model demonstrated superior performances, achieving an AUROC of 0.898 for on-time prediction and an AUROC of 0.897 for 48h in-advance prediction. The proposed GRU-D++, designed to consider temporal information and missing values, stands out for its predictive accuracy. Our findings suggest that this model can significantly alleviate the workload of medical staff and prevent the worsening of patient conditions by enabling timely interventions for PUs in the ICU.

## 1. Introduction

Pressure ulcers (PUs) are prevalent skin injuries in patients who remain immobile or cannot change positions for extended periods. PUs arise due to prolonged pressure on bony prominences, such as the back of the head, shoulders, elbows, and heels, or from blood circulation disorders [1,2,3]. In the early stages of PUs, simple interventions like wound dressings or patient repositioning are required. However, in advanced cases, the patient’s condition may deteriorate significantly, and surgical procedures are required. Such interventions increase medical expenses [4,5] and the workload of the intensive care unit (ICU) staff [6].

In the ICU, various risk assessment tools, including the Braden scale, Gosnell scale, Norton scale, and Waterlow score, are used to gauge PU risks [7,8,9,10,11,12]. Recently, machine learning (ML)-based PU prediction systems have been developed [13,14,15,16,17,18] to effectively predict PU occurrences. These systems employ logistic regression, random forest, boosting machines, or multi-layer perceptron. However, they suffer two major problems: (1) they cannot handle time series data even though real-world data are time series and (2) they cannot appropriately handle missing values even though real-world data contain a massive number of missing values.

To overcome these problems, we develop recurrent neural network (RNN)-based PU prediction systems. RNN is a deep learning (DL) method for time series data. We employ five different RNNs. Simple RNN, gated recurrent unit (GRU) [19], and long-short-term memory (LSTM) [20] are representative RNN models. However, they are not suitable for missing-value handling. Therefore, we also employ GRU with decay (GRU-D) [21], which is specialized to impute missing values. Furthermore, we propose an enhanced version of GRU-D named GRU-D++, suitable for time series with a high missing-value rate.

In the empirical experiments, Simple RNN, GRU, and LSTM outperform traditional ML systems, which indicates the importance of time series information for PU prediction. GRU-D and GRU-D++ outperform RNN systems, demonstrating that their missing-value imputation mechanisms are very effective. In addition, we conduct additional experiments of GRU-D++ with different numbers of input variables, data rescaling, and model finetuning. These experiments can provide meaningful insights to researchers who want to employ GRU-D++ in other medical centers. For the reproducibility of our experiments, we have publicly opened the source code of GRU-D++ at https://github.com/gim4855744/GRU-Dpp accessed on 6 November 2023.

## 2. Methods

### 2.1. Study Population

We used the Medical Information Mart for Intensive Care IV (MIMIC-IV) [22], a large public database containing de-identified patient information admitted to the Beth Israel Deaconess Medical Center (BIDMC), as the internal dataset. Note that, although the MIMIC-IV is a public database, the use of full MIMIC-IV requires approval from PhysioNet. Only ICU patients were included in this study. We classified patients who had records of PUs or who had been assigned PU grades in the nursing records as the PU group. The remaining patients were classified as the non-PU group. Patients with PUs before ICU admissions were excluded from this study.

The external validation dataset was collected from the Kangwon National University Hospital (KNUH) in the Republic of Korea. We used adult patients (age ≥ 18) who were admitted to the ICU between January 2016 and August 2022 and had at least 48 h of records after admission. The dataset has been approved by the Institutional Review Board of KNUH (IRB, KNUH-2022-09-013-00).

### 2.2. Data Collection

**Figure 1:**
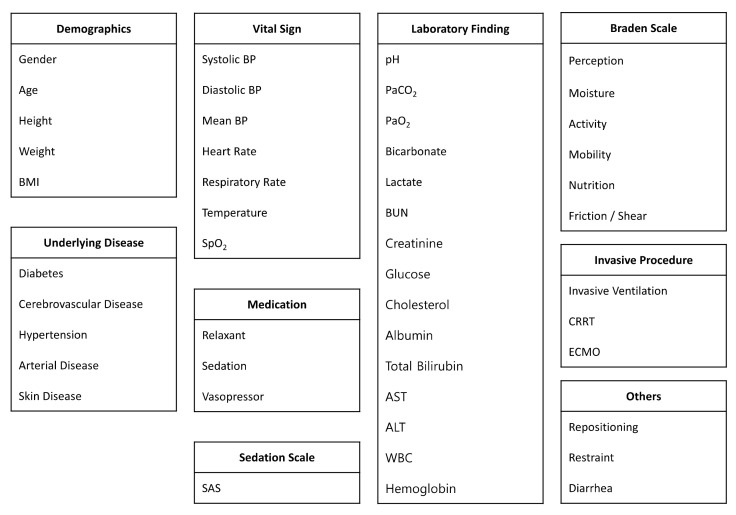
List of input variables.

We used 48 variables. Our input variables included patient demographics, vital signs, laboratory findings, medication and treatment information, underlying diseases, the Braden scales, and the sedation scale. In the MIMIC-IV, some patients have the Richmond-Agitation Sedation Scale (RASS), and the others have the Riker Sedation-Agitation Scale (SAS). Therefore, we converted RASS to SAS. Figure 1 shows the list of our input variables. In this study, we use real-world datasets collected hourly. Therefore, some of the variables have large amounts of missing values; for instance, the laboratory events pH and lactate are missing 72% and 80%, respectively, whereas vital signs such SBP, DBP, and MBP are 0.6% missing.

### 2.3. Data Preprocessing

We performed the following preprocessing steps to prepare patients’ time series:

- We sampled patient data hourly from ICU admission to discharge;
- If multiple sampling data exist in an hour, we selected the average values for continuous variables, the most negative values for categorical variables, and binarized medication information;
- We applied the interquartile range method to discard outliers.

**Figure 2:**
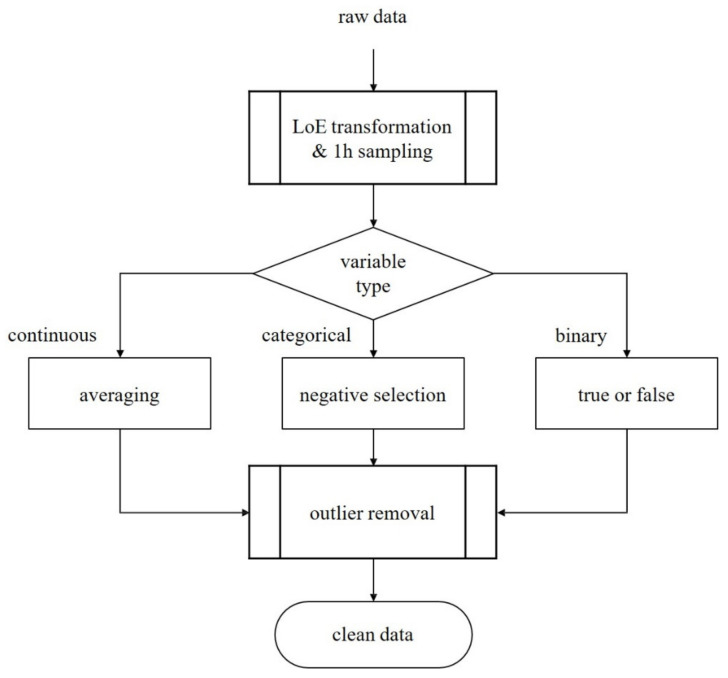
Flow chart of the preprocessing steps.

Figure 2 displays a flow chart of the preprocessing steps.

The input variables of a dataset have distinct scales, which is problematic for effectively training ML models. Thus, we performed min–max scaling with the range [−1, 1]. We performed forward filling and then mean filling to impute missing values. This imputation was not performed when using GRU-D and GRU-D++ because they automatically impute missing values internally.

### 2.4. Prediction Models

In this study, we compare nine ML and DL models. Logistic regression (LR), decision tree (DT), random forest (RF), and extreme gradient boosting machine (XGBoost) are representative machine learning models that have been widely used in previous studies. However, they can only take 1 h of data as input and cannot capture time-varying information. Therefore, they are not suitable to handle real-time data.

RNN, GRU, and LSTM are deep learning models for time series data. Since they take information from the previous time step as input again, they can capture time-varying information. Due to this advantage, they are widely used in real-time applications. However, the missing value problem is another challenge in the medical domain. Real-world electronic health records (EHR) contain many missing values, and missing values must be imputed appropriately before being input into a machine learning model.

GRU-D automatically imputes missing values internally and has demonstrated good performance in EHR datasets compared to other ML and DL models with traditional imputation methods such as mean fill. This result indicates that the imputation mechanism of GRU-D is effective. However, we found that GRU-D still has a problem in high missing-value rate datasets. GRU-D requires the last observation of a variable to generate an imputation value. However, the last observation is unavailable when the first value of a variable is missing.

In recent years, various missing value imputation methods for multivariate time series have been proposed [23,24,25,26,27]. Notably, many of these methods adopt a two-phase learning process [23,24,25,26,27], wherein the entire dataset is first imputed using an imputation model, followed by the training of a classification model. However, this two-phase approach is time-consuming for both training and inference. Some recent methods utilize generative adversarial networks (GANs) [24,25,26], but it is well known that training GANs is notoriously difficult.

**Figure 3:**
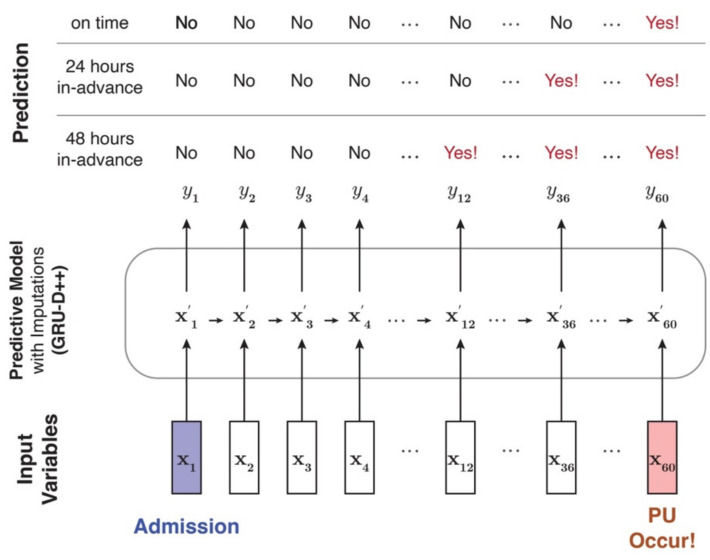
Overview of the proposed in-advance PU prediction system. The blue cell indicates the patient’s admission time, and the red cell indicates the PU occurrence time. Here, x_t_ represents the input variables updated every hour, and x′_t_ represents imputed complete variables. Furthermore, y_t_ is the output of whether PU occurs or not, which was used for training and evaluating our model. The definition of y_t_ can be changed according to the in-advance prediction setting. For example, in 24 h of in-advance prediction, if PU occurs 60 h after admission, the first “yes” should be y_36_.

To overcome this problem, we developed a novel deep learning model named GRU-D++ in this study. Our GRU-D++ is an end-to-end (training and inference of imputation and classification are simultaneously performed) and RNN-based model, practical in real-world scenarios. In addition, we focused on making GRU-D++ able to handle high missing-rate datasets effectively. To the best of our knowledge, GRU-D++ is the first model that explicitly considers high missing-rate data. Details of GRU-D++ can be found in Appendix A. Figure 3 depicts the overview of our proposed real-time in-advance PU prediction system.

## 3. Results

### 3.1. Baseline Characteristics

The internal cohort, MIMIC-IV, consists of 67,175 patients, whereas the external cohort, KNUH, consists of 6876 patients. In the internal cohort, we used 53,740 patients for training and 13,435 for validation. Table 1 displays the baseline characteristics of both the internal and external cohorts.

### 3.2. Predictive Performances

We used the area under the receiver operating characteristics curve (AUROC) and the area under the precision–recall curve (AUPRC) to evaluate the experimental models. AUROC is a common evaluation metric for binary classification. AUPRC is a similar metric to AUROC, but it considers class imbalance. We not only evaluated the performances at the PU occurrence time but also assessed early prediction performances (i.e., 12, 24, and 48 h in-advance predictions).

Table 2 presents the performances of the experimental models on the internal validation set. The results indicated that DT performed poorly with an AUROC of 0.569 at PU occurrence time, whereas the other ML models, LR, RF, and XGBoost exhibited higher performances with AUROCs of 0.818, 0.818, and 0.814 at the PU occurrence time. All RNN models exhibited higher performances than the ML models. In particular, GRU exhibited an AUROC of 0.918 at the PU occurrence time. GRU-D outperformed other RNNs. Furthermore, GRU-D++ exhibited the best performances in all experimental settings, indicating the superiority of its imputation mechanism.

We evaluated the performance of the Braden scale, which is widely used to measure PU risks in the ICU. The AUROC of the Braden score showed lower results at all times compared to the AI model (Table 3).

**Figure 4:**
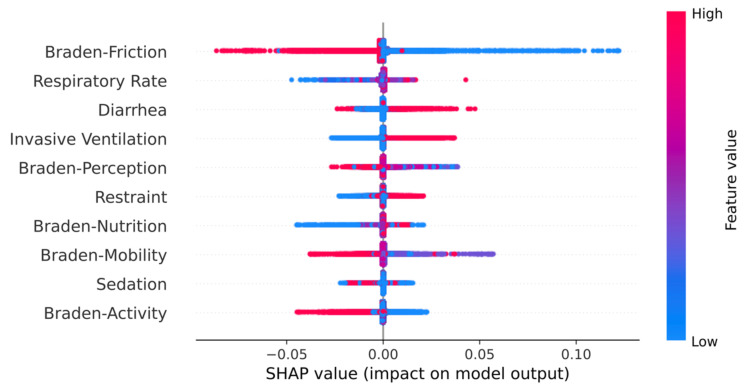
SHAP values of top ten influential variables.

We used 48 variables in the experiment, but these variables may not be available in other medical centers. Since the utilizability of the prediction system is essential, we trained and evaluated GRU-D++ again using the top ten variables, which have the highest SHAP values. The top ten variables are displayed in Figure 4. In Table 3, GRU-D++^10^ indicates GRU-D++ with the top ten variables. GRU-D++^10^ outperformed other RNN models trained with 48 variables, even though it only uses ten variables. Additionally, we conducted the GRU-D++ with 42 variables out of 48 variables, excluding the six variables corresponding to the Braden scales, and the results were presented on GRU-D++ (w/o Braden) in Table 3.

Table 4 shows the performances of GRU-D++ on the external validation set. GRU-D++ achieved a promising performance (an AUROC of 0.807 at the PU occurrence time). To improve the performance of GRU-D++ on the external validation set, we rescale the external set to have a similar distribution to the internal set. Consequently, GRU-D++ (rescale) exhibited a significantly higher performance than GRU-D++ (AUROC 0.895 vs. 0.807 at the PU occurrence time). In addition, we evaluated the performance of GUR-D++ after fine-tuning. Specifically, we randomly split the external set into training (10%), model selection (10%), and validation (80%) sets. As a result, GRU-D++ (fine-tune) exhibited the highest performances on the external set. A comparison of all experimental models on the external set is presented in Appendix A.

## 4. Discussion

### 4.1. Analysis of Predictive Performances

We conducted extensive experiments using various ML and DL models to predict PU occurrences. DT exhibited poor predictive performance because the model is susceptible to overfitting, that is, it cannot accurately predict unseen data. By contrast, other baseline models, LR, RF, and XGBoost, exhibited good performances because of their strong regularization abilities. This is a well-known phenomenon, and previous studies have demonstrated that XGBoost has good predictive power.

The RNN models, Simple RNN, GRU, and LSTM, demonstrated considerably higher performances than the ML models, highlighting the importance of considering time-varying information. Many researchers still widely use boosting machine for their studies, but we showed that RNN models are very helpful for time series data. In addition, GRU and LSTM significantly outperformed Simple RNN, which is unsuitable for capturing long-term temporal information. This result suggests that patients’ long-term information is important in predicting PU occurrences. GRU outperformed LSTM, which is a well-known phenomenon. LSTM requires more data samples than GRU and is susceptible to the overfitting problem.

GRU-D and GRU-D++ significantly outperformed the other ML and RNN models, demonstrating that conventional imputation techniques are insufficient to handle missing values and that the trainable imputation methods of GRU-D and GRU-D++ are effective. In addition, GRU-D++ outperformed GRU-D, which indicates that GRU-D++ is more effective in handling high missing-value rate datasets than GRU-D. We also evaluated GRU-D++ with fewer variables (i.e., ten of the highest SHAP scores) and found that it outperformed the other RNNs with 48 variables. This is an important finding because other medical centers may be unable to collect all 48 variables.

We evaluated GRU-D++ on the external validation set and determined that it exhibited good predictive performances (AUROC >0.8). An issue with directly applying GRU-D++ trained with the internal validation set to the external validation set is that the imputation mechanism of GRU-D++ was trained to predict the internal validation set accurately. However, the distributions of the internal and external validation sets may differ. Thus, the imputation mechanism may fail to generate accurate imputation values on the external validation set, which can result in a deterioration in the predictive performance of GRU-D++. To address this problem, we rescaled the external validation set to have a distribution similar to the internal validation set as much as possible. GRU-D++ with rescaling exhibited a considerably improved performance over basic GRU-D++, demonstrating that GRU-D++ can achieve predictive performances close to those of the internal validation set on any other external sets (e.g., datasets from other medical centers) via rescaling. Furthermore, we retrained (fine-tuned) GRU-D++ with a small part of the external validation set. It demonstrated superior predictive performance with a few data samples and is effective for other medical centers.

### 4.2. Clinical Findings

Similar to previous studies, we developed PU prediction systems using various ML and DL models, classifying data into PU and non-PU groups. However, although previous studies [3,8] have predicted PU occurrences using average values of each variable as input, we used time series data to predict PU occurrences. This study overcomes the performance degradation problem of existing studies using time series information and a novel missing value imputation mechanism. Our system is very effective in real-time in-advance prediction.

The MIMIC-IV dataset used for model development has been widely used in various studies because of its diverse variables. However, the dataset has a disproportionate representation, with Whites and Blacks accounting for approximately 78.56% and Asians making up only approximately 2.94% of the total dataset. Due to this racial imbalance, applying the results of studies only performed with the MIMIC-IV to Asians is inappropriate. One advantage of this study is presenting a prediction system suitable for diverse ethnicities by validating the system developed using the MIMIC-IV with Asian data (KNUH).

An analysis of the SHAP values revealed that, as expected, the Braden scores were among the top ten essential variables for predicting PU occurrences. Interestingly, PU prediction using only 42 variables without the Braden score demonstrated only 1.2–1.4% degradation in AUROC to that with 48 variables (Table 3). This finding indicates that the 42 variables contain valuable information about PU occurrences. Furthermore, using GRU-D++ allows for accurate predictions without the Braden scales, given that these scales inherently have been measured based on the patient’s condition related to the 42 variables. Clinically, this could potentially reduce the workload of nurses who measure and record the Braden score. We anticipate that this model could be a valuable tool for future clinical use.

## 5. Conclusions

PU adversely affects patient health and increases the workload of medical staff in the ICU. Therefore, early prediction of PU is crucial. This study compared various ML and DL models to develop accurate PU prediction systems. As a result, we developed the GRU-D++ model, which covers a high missing-value rate. This model demonstrated better performance than the Braden scale, and it even showed high performance when implemented without the Braden scale. It is expected to contribute significantly to reducing the workload of medical staff in the future.

GRU-D++ can be helpful to other researchers aiming to predict PU occurrences accurately. Furthermore, in future work, we plan to develop a system that integrates the precise prediction of PU occurrence region and grade predictions for PU.

## Figures and Tables

**Table 1:** Baseline characteristics.

Variables	MIMIC-IV (Internal)						KNUH (External)		
	Development Cohort			Validation Cohort			Validation Cohort		
	Control (*n* = 51,600)	Pressure Ulcer (*n* = 2140)	*p*-Value	Control (*n* = 12,876)	Pressure Ulcer (*n* = 559)	*p*-Value	Control (*n* = 6317)	Pressure Ulcer (*n* = 559)	*p*-Value
**Demographics**									
Male, *n* (%)	28,600 (55.43%)	1265 (59.11%)	<0.001	7125 (55.34%)	352 (62.97%)	<0.001	1575 (44.82%)	164 (65.86%)	<0.001
Age *	63.74 (±17.10)	65.44 (±15.22)	<0.001	63.76 (±16.89)	64.98 (±16.25)	0.096	61.32 (±20.90)	72.60 (±13.08)	<0.001
Height (cm) *	168.56 (±9.08)	169.14 (±10.34)	0.004	168.49 (±9.07)	169.52 (±10.52)	0.009	161.75 (±8.57)	162.32 (±9.39)	0.314
Body Weight (kg) *	81.92 (±35.39)	85.49 (±28.56)	<0.001	81.98 (±27.48)	83.94 (±24.21)	0.097	61.89 (±14.38)	56.78 (±14.33)	<0.001
BMI *	28.79 (±13.01)	29.79 (±9.35)	<0.001	28.84 (±9.44)	29.12 (±7.59)	0.481	23.61 (±4.88)	21.46 (±4.77)	<0.001
**Underlying Disease**									
DM, *n* (%)	15,243 (29.54%)	728 (34.02%)	<0.001	3661 (28.44%)	192 (34.35%)	0.003	221 (3.50%)	38 (6.80%)	<0.001
CVD, *n* (%)	8236 (15.96%)	465 (21.730%)	<0.001	2019 (15.68%)	118 (21.11%)	<0.001	793 (12.55%)	111 (19.86%)	<0.001
Hypertension, *n* (%)	32,541 (63.06%)	1385 (64.72%)	0.125	8081 (62.76%)	374 (66.91%)	0.052	141 (2.23%)	10 (1.79%)	0.593
Arterial Disease, *n* (%)	5958 (11.55%)	385 (17.99%)	<0.001	1496 (11.62%)	100 (17.89%)	<0.001	71 (1.12%)	10 (1.79%)	0.233
Skin Disease, *n* (%)	4852 (9.40%)	510 (23.83%)	<0.001	1235 (9.59%)	136 (24.33%)	<0.001	101 (1.60%)	55 (9.84%)	<0.001

*: mean and standard deviation, DM: diabetes mellitus, CVD: cerebrovascular disease, BMI: body mass index.

**Table 2:** Performance comparison of various models on the internal validation set.

	On Time		12 h In Advance		24 h In Advance		48 h In Advance	
	AUROC	AUPRC	AUROC	AUPRC	AUROC	AUPRC	AUROC	AUPRC
LR	0.818 (±0.000)	0.433 (±0.001)	0.814 (±0.000)	0.438 (±0.001)	0.810 (±0.000)	0.442 (±0.001)	0.804 (±0.000)	0.450 (±0.001)
DT	0.569 (±0.007)	0.145 (±0.006)	0.567 (±0.007)	0.151 (±0.005)	0.566 (±0.005)	0.156 (±0.004)	0.569 (±0.004)	0.172 (±0.003)
RF	0.818 (±0.000)	0.407 (±0.005)	0.813 (±0.001)	0.412 (±0.004)	0.808 (±0.001)	0.414 (±0.004)	0.801 (±0.001)	0.424 (±0.004)
XGBoost	0.814 (±0.002)	0.404 (±0.006)	0.807 (±0.003)	0.404 (±0.006)	0.805 (±0.006)	0.410 (±0.012)	0.797 (±0.003)	0.424 (±0.011)
Simple RNN	0.860 (±0.021)	0.489 (±0.037)	0.864 (±0.003)	0.506 (±0.015)	0.859 (±0.010)	0.511 (±0.022)	0.837 (±0.003)	0.507 (±0.015)
GRU	0.918 (±0.003)	0.657 (±0.011)	0.913 (±0.002)	0.657 (±0.009)	0.905 (±0.001)	0.649 (±0.008)	0.885 (±0.003)	0.630 (±0.007)
LSTM	0.909 (±0.005)	0.625 (±0.020)	0.903 (±0.004)	0.619 (±0.019)	0.900 (±0.007)	0.620 (±0.020)	0.880 (±0.005)	0.605 (±0.013)
GRU-D	0.944 (±0.003)	0.737 (±0.008)	0.938 (±0.002)	0.723 (±0.009)	0.930 (±0.003)	0.712 (±0.005)	0.909 (±0.002)	0.686 (±0.007)
GRU-D++	**0.945 (±0.003)**	**0.742 (±0.005)**	**0.940 (±0.002)**	**0.730 (±0.004)**	**0.933 (±0.001)**	**0.722 (±0.005)**	**0.912 (±0.003)**	**0.699 (±0.007)**

LR: logistic regression, DT: decision tree, RF: random forest, XGBoost: extreme gradient boosting machine, RNN: recurrent neural network, GRU: grated recurrent unit, LSTM: long short-term memory, GRU-D: gated recurrent unit with a decay.

**Table 3:** Performance comparison of different input variables on the internal validation set.

	On Time		12 h In Advance		24 h In Advance		48 h In Advance	
	AUROC	AUPRC	AUROC	AUPRC	AUROC	AUPRC	AUROC	AUPRC
Braden scale	0.730	0.201	0.730	0.212	0.731	0.223	0.732	0.245
GRU-D++	**0.945 (±0.003)**	**0.742 (±0.005)**	**0.940 (±0.002)**	**0.730 (±0.004)**	**0.933 (±0.001)**	**0.722 (±0.005)**	**0.912 (±0.003)**	**0.699 (±0.007)**
GRU-D++^10^	0.923 (±0.004)	0.670 (±0.012)	0.918 (±0.003)	0.665 (±0.004)	0.911 (±0.005)	0.658 (±0.003)	0.888 (±0.007)	0.637 (±0.008)
GRU-D++ (w/o Braden)	0.934 (±0.002)	0.671 (±0.009)	0.928 (±0.002)	0.660 (±0.010)	0.920 (±0.001)	0.648 (±0.010)	0.901 (±0.002)	0.631 (±0.011)

GRU-D++^10^: GRU-D++ trained with top ten important variables, GRU-D++ (w/o braden): GRU-D++ trained without the Braden scales.

**Table 4:** Performances of GRU-D++ on the external validation set.

	On Time		12 h In Advance		24 h In Advance		48 h In Advance	
	AUROC	AUPRC	AUROC	AUPRC	AUROC	AUPRC	AUROC	AUPRC
GRU-D++	0.807 (±0.015)	0.350 (±0.026)	0.827 (±0.018)	0.380 (±0.031)	0.826 (±0.035)	0.398 (±0.054)	0.848 (±0.016)	0.445 (±0.030)
GRU-D++ (rescale)	0.895 (±0.032)	0.534 (±0.078)	0.892 (±0.022)	0.538 (±0.044)	0.889 (±0.017)	0.539 (±0.049)	0.864 (±0.017)	0.514 (±0.023)
GRU-D++ (fine-tune)	0.898 (±0.018)	0.523 (±0.051)	0.901 (±0.016)	0.542 (±0.045)	0.895 (±0.019)	0.543 (±0.050)	0.897 (±0.008)	0.575 (±0.027)

**Figure A1:**
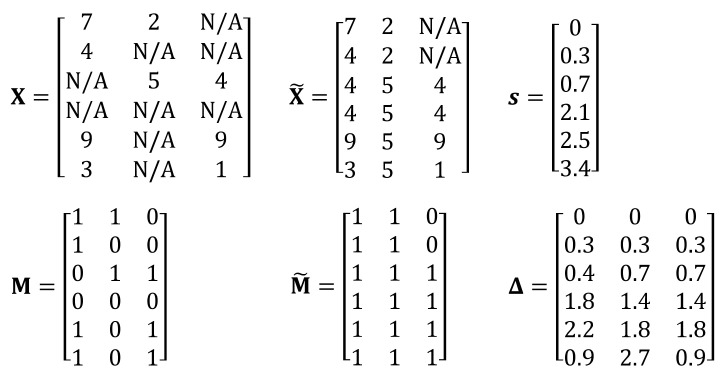
An example of $X,\tilde{X},M,\tilde{M},s,\;\mathrm{and}\;\;\Delta$. This time series has three variables and six time steps. N/A = not applicable.

## Data Availability

Data sharing is possible via the approval process of the Data Review Committee of Kangwon National University Hospital.

## Appendix A

### Appendix A.1. Details of GRU-D++

Having missing values in real-world multivariate time series causes poor prediction results. To address this problem, many studies have been conducted to impute missing values [25,26,27]. GRU-D [23] can directly handle missing values. GRU-D implicitly learns the weighted sum between the means and last observations of input variables. It outperforms RNNs with conventional imputation methods on the MIMIC-III and PhysioNet datasets, which are real-world electronic health records. However, GRU-D is still not suitable for high missing-value rate datasets. To overcome this limitation, we proposed a novel GRU-based model for missing values called GRU-D++ in this study.

Let $X=\left(x_{1},x_{2},\ldots ,x_{T}\right)\in {\mathbb{R}}^{T\times M}$ be a multivariate time series, where T is the number of time steps and M is the number of input variables. Given a multivariate time series X, we define a mask $M\in {\left\{0,1\right\}}^{T\times M}$ for X as follows:

(A1) $$\mu_{t,m}=\left\{\begin{array}{c} 0,\;\;\;\mathrm{if}\;x_{t,m}\;\mathrm{is}\;N/A\\ 1,\;\;\;\mathrm{otherwise} \end{array}\right.\in M$$

where N/A indicates a missing value. Then, the last observation $\tilde{X}\in {\mathbb{R}}^{T\times M}$ for X and the corresponding mask $\tilde{M}\in {\left\{0,1\right\}}^{T\times M}$ are defined as follows:

(A2) $${\tilde{x}}_{t,m}=\left\{\begin{array}{c} \;\;x_{t,m},\;\;\;\;\mathrm{if}\;\mu_{t,m}=1\\ {\tilde{x}}_{t-1,m},\;\;\;\mathrm{if}\;\mu_{t,m}=0\;\mathrm{and}\;t>1\\ \;\;N/A,\;\;\;\mathrm{otherwise} \end{array}\right.\in \tilde{X}$$

(A3) $${\tilde{\mu }}_{t,m}=\left\{\begin{array}{c} 0,\;\;\;\mathrm{if}\;{\tilde{x}}_{t,m}\;\mathrm{is}\;N/A\\ 1,\;\;\;\mathrm{otherwise} \end{array}\right.\in M$$

We then record time intervals $\Delta \in {\mathbb{R}}^{T\times M}$ as follows:

(A4) $$\delta_{t,m}=\left\{\begin{array}{c} x_{t,m}-s_{t-1}+\delta_{t-1,m},\;\;\mathrm{if}\;t>1\;\mathrm{and}\;\mu_{t-1,m}=0\\ \;\;\;x_{t,m}-s_{t-1},\;\;\mathrm{if}\;t>1\;\mathrm{and}\;\mu_{t-1,m}=1\in \tilde{X}\\ \;\;\;\;\;\;0,\;\;\;\mathrm{otherwise} \end{array}\right.$$

where $s=\left(s_{1},s_{2},\ldots ,s_{T}\right)\in {\mathbb{R}}^{T}$ indicates the timestamps for X. (A4) is the same as the method detailed in [23]. Figure A1 illustrates an example of X, $\tilde{X}$, M, $\tilde{M}$, s, and Δ.

Because time intervals between each time step can be different, we applied a trainable soft time decaying mechanism to input variables and hidden states of GRU-D++. The decaying weight vector $\gamma_{x_{t}}$ and $\gamma_{h_{t}}$ for the input $x_{t}$ and hidden state $h_{t}$ are defined as follows:

(A5) $$\gamma_{x_{t}}=\exp\left(-\max\left(0,\delta_{t}W_{x_{t}}+b_{x_{t}}\right)\right)⊙{\tilde{\mu }}_{t},$$

(A6) $$\gamma_{h_{t}}=\exp\left(-\max\left(0,\delta_{t}W_{h_{t}}+b_{h_{t}}\right)\right),$$

where $W_{x_{t}}\in {\mathbb{R}}^{M\times M}$, $W_{h_{t}}\in {\mathbb{R}}^{M\times K}$, $b_{x_{t}}\in {\mathbb{R}}^{M}$, and $b_{h_{t}}\in {\mathbb{R}}^{K}$ are trainable weights and biases. Unlike GRU-D, $W_{x_{t}}$ is a dense matrix, and $\gamma_{x_{t},m}$ is held to 0 if the last observation ${\tilde{\mu }}_{t,m}$ is missing. These decaying mechanisms help GRU-D++ capture temporal dynamics in various time intervals. The trainable imputation method is defined as follows:

(A7) $$x_{t,m}^{\prime }=m_{t,m}\;x_{t,m}+\left(1-m_{t,m}\right)\times \left(\gamma_{x_{t},m}\;{\tilde{x}}_{t,m}+\left(1-\gamma_{x_{t},m}\right)\;{\bar{x}}_{m}\right)$$

where ${\bar{x}}_{m}$ indicates the arithmetic mean of the m^th^ variable. Finally, GRU-D++ is defined as follows:

(A8) $$h_{t-1}^{\prime }=\gamma_{h_{t}}h_{t-1},$$

(A9) $$r_{t}=\sigma \left(x_{t}^{\prime }W_{r}+h_{t-1}^{\prime }U_{r}+m_{t}V_{r}+{\tilde{m}}_{t}Q_{r}+b_{r}\right),$$

(A10) $$z_{t}=\sigma \left(x_{t}^{\prime }W_{z}+h_{t-1}^{\prime }U_{z}+m_{t}V_{z}+\tilde{m}Q_{z}+b_{z}\right),$$

(A11) $${\tilde{h}}_{t}=\tanh\left(x_{t}^{\prime }W+\left(r_{t}⊙h_{t-1}^{\prime }\right)U+m_{t}V+\tilde{m}Q+b\right),$$

(A12) $$h_{t}=\left(1-z_{t}\right)⊙h_{t-1}^{\prime }+z_{t}⊙{\tilde{h}}_{t}.$$

### Appendix A.2. Further Experiments

In this section, we present a comparison of all experimental models on the external validation set. Table A1 displays the performances of the experimental models on the external validation set, where GRU outperforms GRU-D++. The imputation mechanisms of GRU-D and GRU-D++ were trained to predict the internal set accurately. Therefore, they may fail to generate precise imputation values on the external set because the distributions of the internal and external sets may differ. Consequently, GRU-D and GRU-D++ exhibit lower performances than GRU. To address this problem, we rescaled the external validation set to have a similar distribution to the internal set. Table A2 presents the performances of the experimental models after rescaling, and a considerable improvement in the GRU-D++ performance. Furthermore, we evaluated the performances of RNNs on the external validation set after fine-tuning. Thus, we retrained the RNNs with a small subset of the external set and evaluated the performances on the remaining set. Table A3 demonstrates the performances of the RNNs on the external set after fine-tuning, where GRU-D++ consistently outperforms the other experimental models in all settings.

**Table A1:** Performance comparison on the external validation set.

	On Time		12 h In Advance		24 h In Advance		48 h In Advance	
	AUROC	AUPRC	AUROC	AUPRC	AUROC	AUPRC	AUROC	AUPRC
LR	0.653 (±0.008)	0.212 (±0.002)	0.655 (±0.008)	0.220 (±0.002)	0.656 (±0.008)	0.229 (±0.003)	0.667 (±0.008)	0.249 (±0.003)
DT	0.504 (±0.015)	0.125 (±0.005)	0.502 (±0.037)	0.131 (±0.010)	0.519 (±0.012)	0.141 (±0.006)	0.520 (±0.014)	0.153 (±0.009)
RF	0.652 (±0.011)	0.195 (±0.010)	0.631 (±0.038)	0.203 (±0.016)	0.645 (±0.029)	0.215 (±0.012)	0.662 (±0.033)	0.237 (±0.016)
XGBoost	0.603 (±0.025)	0.189 (±0.010)	0.605 (±0.022)	0.199 (±0.015)	0.627 (±0.026)	0.221 (±0.018)	0.635 (±0.016)	0.233 (±0.015)
RNN	0.768 (±0.033)	0.290 (±0.027)	0.753 (±0.028)	0.295 (±0.024)	0.759 (±0.023)	0.309 (±0.025)	0.751 (±0.008)	0.315 (±0.006)
GRU	**0.880 (±0.015)**	**0.469 (±0.035)**	**0.875 (±0.006)**	**0.471 (±0.025)**	**0.873 (±0.010)**	**0.480 (±0.031)**	**0.867 (±0.008)**	**0.490 (±0.024)**
LSTM	0.816 (±0.042)	0.370 (±0.054)	0.833 (±0.013)	0.390 (±0.027)	0.829 (±0.017)	0.405 (±0.029)	0.815 (±0.027)	0.409 (±0.041)
GRU-D	0.762 (±0.019)	0.267 (±0.015)	0.768 (±0.015)	0.285 (±0.009)	0.777 (±0.022)	0.307 (±0.021)	0.759 (±0.026)	0.291 (±0.029)
GRU-D++	0.807 (±0.015)	0.350 (±0.026)	0.827 (±0.018)	0.380 (±0.031)	0.826 (±0.035)	0.398 (±0.054)	0.848 (±0.016)	0.445 (±0.030)

**Table A2:** Performance comparison on the external validation set with rescaling.

	On Time		12 h In Advance		24 h In Advance		48 h In Advance	
	AUROC	AUPRC	AUROC	AUPRC	AUROC	AUPRC	AUROC	AUPRC
LR	0.643 (±0.010)	0.217 (±0.004)	0.644 (±0.009)	0.226 (±0.004)	0.646 (±0.010)	0.235 (±0.004)	0.657 (±0.007)	0.255 (±0.004)
DT	0.542 (±0.022)	0.139 (±0.011)	0.503 (±0.060)	0.133 (±0.013)	0.539 (±0.024)	0.151 (±0.011)	0.541 (±0.009)	0.161 (±0.005)
RF	0.513 (±0.051)	0.162 (±0.026)	0.590 (±0.044)	0.197 (±0.023)	0.581 (±0.067)	0.202 (±0.029)	0.555 (±0.073)	0.202 (±0.029)
XGBoost	0.565 (±0.033)	0.179 (±0.016)	0.569 (±0.059)	0.187 (±0.022)	0.555 (±0.039)	0.192 (±0.019)	0.587 (±0.048)	0.218 (±0.016)
RNN	0.755 (±0.043)	0.278 (±0.028)	0.774 (±0.029)	0.321 (±0.025)	0.779 (±0.031)	0.336 (±0.029)	0.749 (±0.011)	0.324 (±0.010)
GRU	0.890 (±0.007)	0.513 (±0.024)	0.882 (±0.003)	0.503 (±0.017)	0.880 (±0.006)	0.515 (±0.015)	**0.874 (±0.005)**	**0.521 (±0.006)**
LSTM	0.834 (±0.014)	0.393 (±0.027)	0.838 (±0.031)	0.394 (±0.048)	0.819 (±0.017)	0.390 (±0.026)	0.811 (±0.028)	0.398 (±0.045)
GRU-D	0.824 (±0.045)	0.370 (±0.088)	0.840 (±0.039)	0.407 (±0.070)	0.810 (±0.035)	0.378 (±0.050)	0.726 (±0.083)	0.289 (±0.103)
GRU-D++	**0.895 (±0.032)**	**0.534 (±0.078)**	**0.892 (±0.022)**	**0.538 (±0.044)**	**0.889 (±0.017)**	**0.539 (±0.049)**	0.864 (±0.017)	0.514 (±0.023)

**Table A3:** Performance comparison on the external validation set with finetuning.

	On Time		12 h In Advance		24 h In Advance		48 h In Advance	
	AUROC	AUPRC	AUROC	AUPRC	AUROC	AUPRC	AUROC	AUPRC
RNN	0.786 (±0.014)	0.307 (±0.017)	0.773 (±0.032)	0.318 (±0.019)	0.766 (±0.012)	0.324 (±0.021)	0.771 (±0.012)	0.338 (±0.005)
GRU	0.873 (±0.011)	0.458 (±0.029)	0.872 (±0.008)	0.466 (±0.024)	0.868 (±0.007)	0.473 (±0.026)	0.864 (±0.007)	0.485 (±0.023)
LSTM	0.826 (±0.032)	0.375 (±0.047)	0.832 (±0.026)	0.398 (±0.025)	0.838 (±0.020)	0.414 (±0.036)	0.811 (±0.022)	0.399 (±0.036)
GRU-D	0.858 (±0.015)	0.426 (±0.046)	0.853 (±0.023)	0.424 (±0.044)	0.856 (±0.017)	0.442 (±0.035)	0.845 (±0.018)	0.438 (±0.029)
GRU-D++	**0.898 (±0.018)**	**0.523 (±0.051)**	**0.901 (±0.016)**	**0.542 (±0.045)**	**0.895 (±0.019)**	**0.543 (±0.050)**	**0.897 (±0.008)**	**0.575 (±0.027)**

**Table A4:** Performance comparison on the external validation set without Braden scales.

	On Time		12 h In Advance		24 h In Advance		48 h In Advance	
	AUROC	AUPRC	AUROC	AUPRC	AUROC	AUPRC	AUROC	AUPRC
LR	0.707 (±0.002)	0.234 (±0.001)	0.709 (±0.002)	0.244 (±0.001)	0.711 (±0.002)	0.254 (±0.002)	0.716 (±0.002)	0.274 (±0.002)
DT	0.516 (±0.008)	0.129 (±0.004)	0.513 (±0.008)	0.133 (±0.003)	0.525 (±0.012)	0.143 (±0.006)	0.532 (±0.023)	0.157 (±0.013)
RF	0.683 (±0.004)	0.211 (±0.006)	0.680 (±0.011)	0.217 (±0.007)	0.694 (±0.008)	0.230 (±0.009)	0.693 (±0.004)	0.251 (±0.003)
XGBoost	0.676 (±0.010)	0.220 (±0.009)	0.675 (±0.008)	0.227 (±0.005)	0.683 (±0.013)	0.239 (±0.005)	0.692 (±0.008)	0.264 (±0.005)
RNN	0.761 (±0.014)	0.277 (±0.010)	0.777 (±0.015)	0.290 (±0.009)	0.775 (±0.022)	0.304 (±0.015)	0.772 (±0.006)	0.323 (±0.006)
GRU	**0.856 (±0.010)**	**0.413 (±0.021)**	**0.843 (±0.008)**	**0.406 (±0.015)**	**0.848 (±0.016)**	**0.426 (±0.026)**	**0.837 (±0.027)**	**0.434 (±0.058)**
LSTM	0.844 (±0.020)	0.389 (±0.026)	0.833 (±0.020)	0.388 (±0.043)	0.827 (±0.016)	0.385 (±0.038)	0.813 (±0.010)	0.390 (±0.022)
GRU-D	0.749 (±0.028)	0.270 (±0.029)	0.750 (±0.025)	0.286 (±0.032)	0.761 (±0.025)	0.302 (±0.033)	0.778 (±0.018)	0.344 (±0.030)
GRU-D++	0.783 (±0.033)	0.328 (±0.051)	0.795 (±0.034)	0.337 (±0.046)	0.803 (±0.030)	0.382 (±0.031)	0.818 (±0.034)	0.405 (±0.039)

**Table A5:** Performance comparison on the external validation set without Braden scales after rescaling.

	On Time		12 h In Advance		24 h In Advance		48 h In Advance	
	AUROC	AUPRC	AUROC	AUPRC	AUROC	AUPRC	AUROC	AUPRC
LR	0.697 (±0.003)	0.236 (±0.003)	0.697 (±0.003)	0.245 (±0.003)	0.699 (±0.003)	0.254 (±0.004)	0.702 (±0.003)	0.273 (±0.004)
DT	0.515 (±0.046)	0.132 (±0.019)	0.546 (±0.030)	0.148 (±0.016)	0.537 (±0.032)	0.148 (±0.013)	0.453 (±0.097)	0.138 (±0.016)
RF	0.538 (±0.050)	0.164 (±0.020)	0.612 (±0.042)	0.198 (±0.010)	0.552 (±0.058)	0.185 (±0.016)	0.525 (±0.056)	0.180 (±0.018)
XGBoost	0.537 (±0.057)	0.176 (±0.028)	0.592 (±0.046)	0.197 (±0.024)	0.560 (±0.062)	0.193 (±0.025)	0.568 (±0.050)	0.209 (±0.020)
RNN	0.771 (±0.026)	0.284 (±0.013)	0.761 (±0.016)	0.287 (±0.007)	0.751 (±0.009)	0.301 (±0.004)	0.764 (±0.014)	0.323 (±0.009)
GRU	0.866 (±0.008)	0.446 (±0.022)	0.844 (±0.019)	0.426 (±0.032)	0.850 (±0.021)	0.447 (±0.036)	0.826 (±0.040)	0.439 (±0.062)
LSTM	0.840 (±0.008)	0.380 (±0.010)	0.821 (±0.033)	0.381 (±0.029)	0.798 (±0.039)	0.368 (±0.043)	0.791 (±0.023)	0.376 (±0.021)
GRU-D	0.811 (±0.020)	0.408 (±0.036)	0.853 (±0.013)	0.469 (±0.016)	0.845 (±0.035)	0.468 (±0.048)	0.810 (±0.059)	0.431 (±0.115)
GRU-D++	**0.868 (±0.037)**	**0.492 (±0.078)**	**0.888 (±0.012)**	**0.530 (±0.034)**	**0.889 (±0.014)**	**0.546 (±0.040)**	**0.874 (±0.032)**	**0.543 (±0.062)**

**Table A6:** Performance comparison on the external validation set without Braden scales after finetuning.

	On Time		12 h In Advance		24 h In Advance		48 h In Advance	
	AUROC	AUPRC	AUROC	AUPRC	AUROC	AUPRC	AUROC	AUPRC
RNN	0.771 (±0.015)	0.296 (±0.009)	0.782 (±0.013)	0.315 (±0.007)	0.782 (±0.014)	0.329 (±0.015)	0.784 (±0.001)	0.346 (±0.004)
GRU	0.856 (±0.010)	0.426 (±0.018)	0.844 (±0.011)	0.406 (±0.020)	0.852 (±0.018)	0.436 (±0.029)	0.847 (±0.020)	0.447 (±0.046)
LSTM	0.853 (±0.009)	0.406 (±0.022)	0.851 (±0.012)	0.408 (±0.028)	0.837 (±0.025)	0.405 (±0.025)	0.835 (±0.001)	0.418 (±0.009)
GRU-D	0.852 (±0.010)	0.424 (±0.021)	0.85 (±0.012)	0.439 (±0.031)	0.857 (±0.016)	0.449 (±0.037)	0.845 (±0.006)	0.459 (±0.024)
GRU-D++	**0.881 (±0.025)**	**0.500 (±0.073)**	**0.867 (±0.020)**	**0.475 (±0.059)**	**0.878 (±0.016)**	**0.504 (±0.048)**	**0.875 (±0.011)**	**0.518 (±0.039)**
